# Suberoylanilide hydroxamic acid suppresses hepatic stellate cells activation by HMGB1 dependent reduction of NF-*κ*B1

**DOI:** 10.7717/peerj.1362

**Published:** 2015-11-03

**Authors:** Wenwen Wang, Min Yan, Qiuhong Ji, Jinbiao Lu, Yuhua Ji, Juling Ji

**Affiliations:** 1 Department of Pathology, Medical School of Nantong University, Nantong, China; 2 Department of Pathology, Traditional Chinese Medicine Hospital of Jiangyin City, Jiangyin, China; 3 Neurology Department, Affiliated Hospital of Nantong University, Nantong, China; 4 Key Laboratory of Neuroregeneration, Nantong University, Nantong, China

**Keywords:** Hepatic stellate cell, Histone deacetylase inhibitor, Liver fibrosis, Suberoylanilide hydroxamic acid, Nuclear factor kappa B1, High mobility group box 1

## Abstract

Hepatic stellate cells (HSCs) activation is essential to the pathogenesis of liver fibrosis. Exploring drugs targeting HSC activation is a promising anti-fibrotic strategy. In the present study, we found suberoylanilide hydroxamic acid (SAHA), a histone deacetylase inhibitor, prominently suppressed the activation phenotype of a human hepatic stellate cell line—LX2. The production of collagen type I and *α*-smooth muscle actin (*α*-SMA) as well as the proliferation and migration of LX2 cells were significantly reduced by SAHA treatment. To determine the molecular mechanisms underlying this suppression, genome wild gene regulation by SAHA was determined by Affymetrix 1.0 human cDNA array. Upon SAHA treatment, the abundance of 331 genes was up-regulated and 173 genes was down-regulated in LX2 cells. Bioinformatic analyses of these altered genes highlighted the high mobility group box 1 (HMGB1) pathway was one of the most relevant pathways that contributed to SAHA induced suppression of HSCs activation. Further studies demonstrated the increased acetylation of intracellular HMGB1 in SAHA treated HSCs, and this increasing is most likely to be responsible for SAHA induced down-regulation of nuclear factor kappa B1 (NF-*κ*B1) and is one of the main underlying mechanisms for the therapeutic effect of SAHA for liver fibrosis.

## Introduction

Liver fibrosis is a chronic wound-healing response caused by a variety of injuries such as viral infection, autoimmune and metabolic disease as well as drug or alcoholic induced disease (Pellicoro et al., 2014). It can lead to cirrhosis, and subsequent several life-threatening complications, including portal hypertension, liver failure and hepatocellular carcinoma. Although the underlying mechanisms of liver fibrosis has been extensively studied, we are still in great need of safe and efficient anti-fibrotic drugs (Schuppan & Kim, 2013).

It has been well acknowledged that activation of hepatic stellate cells (HSCs) upon liver injury is the central event that leads to liver fibrosis (Puche, Saiman & Friedman, 2013). Activated HSCs transdifferentiated form vitamin A-storing perisinusoidal cells to proliferative and fibrogenic myofibroblasts, start to express alpha smooth muscle actin (*α*-SMA), produce more extracellular matrix (ECM) and hence cause extensive ECM deposition. A recent fate tracing study confirmed that HSCs gave rise to 82–96% of myofibroblasts in different models of liver disease (Mederacke et al., 2013). Exploring drugs targeting HSCs activation is a promising anti-fibrotic strategy.

Histone deacetylases (HDACs) are primarily identified as enzymes that reverse acetylation of nucleosomal histones on the ε-amino group of lysine residues (Lopez-Rodas et al., 1993). HDACs together with histone lysine acetyltransferases (HATs) are responsible for the balance between acetylated/deacetylated states of histones, therefore transform the chromatin structure and alter gene transcription. Growing number of identified acetylated non-histone proteins demonstrate that reversible lysine acetylation also influence mRNA stability, and the localisation, interaction, degradation and function of non-histone proteins (Choudhary et al., 2009; Spange et al., 2009). HDAC inhibitors (HDACi) generally lead to growth arrest, differentiation and apoptosis of malignant cells, and have been extensively explored as potential anti-cancer agents (West & Johnstone, 2014).

The anti-fibrotic effects of HDACi were first reported by the Geerts lab (Niki et al., 1999). *In vitro* study showed that both sodium butyrate and trichostatin A (TSA) could attenuate rat HSCs activation. The up-regulation of collagen type I and *α*-SMA was blocked and cell proliferation was inhibited upon treatment. TSA also prevented new actin filament formation and reduced HSCs migration (Rombouts et al., 2002). However, the clinical application of TSA is limited, as it can be degraded within 30 min by the hepatocytes (Sanderson et al., 2004).

Suberoylanilide hydroxamic acid (SAHA), a class I and II HDACi, is the first HDACi approved by the Food and Drug Administration (FDA) in the United States for the treatment of cutaneous T-cell lymphoma under the trade name Vorinostat (Duvic et al., 2007). In searching for safe and efficient anti-fibrotic drugs, we found that SAHA, an analog of TSA, prominently suppressed human HSCs activation. Consistently, in a very recent study, Park et al. (2014) reported that SAHA improved liver function, suppressed liver fibrosis and increased survival of bile duct ligation (BDL) rats, accompanied by reduction of cell growth, activation and survival of HSCs. To explore the underlying mechanisms, the genome-wide gene regulation in HSCs by SAHA was determined by cDNA array analyses. By bioinformatic analyses of the altered genes, we found that high mobility group box 1 (HMGB1) pathway was one of the most relevant pathways that contributed to SAHA induced suppression of HSCs activation. Reduced expression and activity of nuclear factor kappa B 1 (NF-*κ*B1) which depended on increased acetylation of HMGB1 might contribute to the suppressive effects of SAHA on HSCs activation.

## Materials and Methods

### Cell culture and IC50 determination

Human hepatic stellate cell line LX2 which resembles activated HSCs (Xu et al., 2005) was maintained in Dulbecco’s modified Eagle’s medium (Gibco, Carlsbad, USA) with 10% fetal bovine serum (Gibco) in an atmosphere of 5% CO_2_ at 37 °C. In order to determine the appropriate concentration of SAHA used in our experiments, the cytotoxicity of SAHA was determined by Cell Counting Kit-8 (CCK8; Beyotime, Nantong, China). Stock solution of 10 mM SAHA were prepared by dissolving 2.643 mg SAHA in 1 ml DMSO. LX2 cells were seeded at 5,000 cells per well in 96-well plate for 24 h, and then treated with SAHA at the concentrations of 0, 0.5, 1.0, 2.5, 5.0 or 10 µM for 0, 24, 36, 48 or 72 h. Control cells were treated with an equal volume of vehicle (0–0.1% DMSO). CCK8 solution was added (10 µl each well) and incubated at 37 °C for 2 h. The optical density readings at 450 nm were determined by a microplate reader (Bio-Rad, Tokyo, Japan). The 50% inhibitory concentration (IC50) of SAHA for the proliferation of LX2 cells was calculated by the intersection of the plotted line.

### Proliferation and migration assay

Forty-eight hours after SAHA treatment, cell proliferation was measured by 5-ethynyl-2′-deoxyuridine (EdU) incorporation assay by EdU assay kit (RiboBio, Guangzhou, China), according to the manufacturer’s instructions. Hoechst 33258 (Beyotime) was used for counter-staining of the nuclei, the cells were visualized under a fluorescent microscope (Olympus, Tokyo, Japan), EdU positive cells (red) were counted using Image Pro Plus 5.0 software (Media Cybernetics, Bethesda, MD). The results were expressed as the labeling index according to the following formula: number of EdU-positive nuclei ×100/number of total nuclei.

For migration assays, 20,000 SAHA treated or untreated LX2 cells resuspended in 200 µl serum-free DMEM were plated in the upper chambers (Millicell, 0.8 µm; Millipore, Bedford, MA), DMEM medium with 2.5% FBS was used as a chemoattractant in the lower chambers. After 16 h, nonmigrating cells were removed from the upper surface softly by a cotton swab. The cells that migrated through the membrane to the lower surface were fixed with 4% paraformaldehyde and stained with 0.5% crystal violet, then counted under a microscope (Olympus) at 200-fold magnification.

### cDNA microarray experiments and bioinformatic analysis

Total RNAs were extracted from LX2 or SAHA treated LX2 cells using TRIzol (Invitrogen, Carlsbad, CA) according to the manufacturer’s instructions. cDNA microarray experiments using GeneChip Human Gene 1.0 ST Array (Affymetrix, Santa Clara, CA) were performed according to the standard Affymetrix protocol, each with biological replica. The BRB Array Tools version 4.3.1 (http://linus.nci.nih.gov/BRB-ArrayTools.html) was used for the analyses of cDNA microarray gene expression data as previously describe (Ji et al., 2015). Class Comparison Tool based on univariate *F*-tests was used to find genes differentially expressed between groups. The canonical pathways of differentially expressed genes were generated using Ingenuity Pathways Analysis (IPA; Ingenuity Systems, Redwood City, CA).

### siRNA transfection

LX2 cells were seeded into 6-well culture plates. Cells in exponential status were transfected with small interfering RNAs (siRNAs) against HMGB1 (siR-HMGB1) (SASI_Hs01_00196036 and SASI_Hs01_00196037, Sigma) using SuperFectin II *in vitro* DNA transfection reagent (Pufei Biotech, Shanghai, China). Non-targeting control siRNA (siR-NC) was used as negative control. Knockdown efficiency was determined by real-time quantitative reverse transcription-PCR (RT-PCR) and western blots (Fig. S1). For some groups, cells were treated with SAHA 48 h after siRNA transfection.

### Real-time quantitative RT-PCR

The RNAs from LX2 or SAHA treated LX2 cells were reverse-transcribed with Thermoscript RT-PCR system (Invitrogen). Real-time quantitative PCR was performed on RotorGene 3000 instrument (Corbett Research, New South Wales, Australia) with FastStart Universal SYBR Green Master kit (Roche, Mannheim, Germany). Specific gene primers were provided in Table S1. The relative gene expression ratios were calculated as 2 − ΔC*τ* values (normalized to house keeping gene GAPDH).

### Western blot, immunoprecipitation and immunofluorescence staining

For western blot analysis, whole cell protein was extracted by RIPA lysis buffer (Beyotime) according to the manufacturer’s instructions. Equal amounts of protein (30 µg) were separated on 10% SDS PAGE gel and transferred onto polyvinyldifluoride (PVDF) membranes (Millipore). PVDF membranes were blocked with 5% non-fat milk for 1 h, then incubated with specific primary antibodies for pan acetyllysine (PTM BioLabs, Hangzhou, China), *α*-smooth muscle actin (*α*-SMA, Abcam, Cambridge, UK), Collagen I (R&D Systems, Minneapolis, MN), NF-*κ*B1 (Santa Cruz Biotechnology, Santa Cruz, CA), HMGB1 (Abcam) and GAPDH (Beyotime) at 4 °C overnight, then incubated with horseradish peroxidase-conjugated secondary antibody for an additional 1 h at room temperature. The protein expression was visualized with the ECL chemiluminescence detection system (Pierce Chemical Co., Rockford, IL).

Immunoprecipitation was performed by incubating 1.0 mg total cell lysates with 1 µg antibody against acetyl-lysine at 4 °C overnight. Normal mouse IgG was used as a negative control, protein A/G agarose (Santa Cruz) was added and incubated for 2 h at 4 °C while rocking. After that, precipitates were washed four times with cold PBS buffer, suspended in 2× SDS buffer (Beyotime) and subjected to western blot analysis.

LX2 cells were cultured on coverslips were fixed with 2% paraformaldehyde for 15 min on ice, followed by incubation with antibody against HMGB1 (Abcam) at 4 °C overnight, Cy3 labeled secondary antibody (Beyotime) for 1 h in dark. Nuclei were counter-stained with 0.1 mg/ml Hoechst 33258 (Beyotime) for 2 min. Slides were mounted with glycerol and photographed with fluorescent microscope (Olympus).

### NF-*κ*B1 luciferase reporter assay

LX2 cells were seeded to 24-well plates (3 × 10^5^ cells per well) 24 h before transfection with NF-*κ*B Firefly luciferase reporter plasmid and pGMR-TK R*enilla* luciferase reporter plasmid (Genomeditech, Shanghai, China). Six hours after transfection, LX2 cells were treated with or without SAHA or transfected with or without si-HMGB1 for 24 h. Cell lysates were prepared using Passive Lysis Buffer (Promega, Madison, WI), *Firefly* and *Renilla* luciferase activities were assessed using a Dual Luciferase Reporter Assay System (Promega) according to the manufacturer’s instructions. *Firefly* luciferase activities were normalized to *Renilla* luciferase, and the activities of NF-*κ*B1 were compared.

### Statistical analysis

Values were expressed as means ± standard deviation (SD). Statistical analyses were carried out using Student’s *t* test or one-way ANOVA analysis (Graphpad Prism 5.0). In IPA analysis, the statistical data were generated by the software. All *P*-values were two-sided and the statistical significance was defined as *P* < 0.05. Unless otherwise specified, all assays were performed in triplicate.

## Results and Discussion

### SAHA treatment attenuated HSCs activation

The IC50 of SAHA for the proliferation of LX2 cells was determined by CCK8 assay. LX2 cells were exposed to different concentrations of SAHA ranging from 0 to 10 µM for 0, 24, 36, 48 or 72 h. Serving as the vehicle, DMSO (up to 0.1%) did not affect the viability or growth of LX2 cell line. SAHA was able to inhibit HSCs proliferation in a dose-dependent manner (Fig. S1). According to the inhibition rate at 48 h, the concentration of 2.5 µM was determined as IC50 (Fig. S1) and was used in the following experiments.

**Figure 1:**
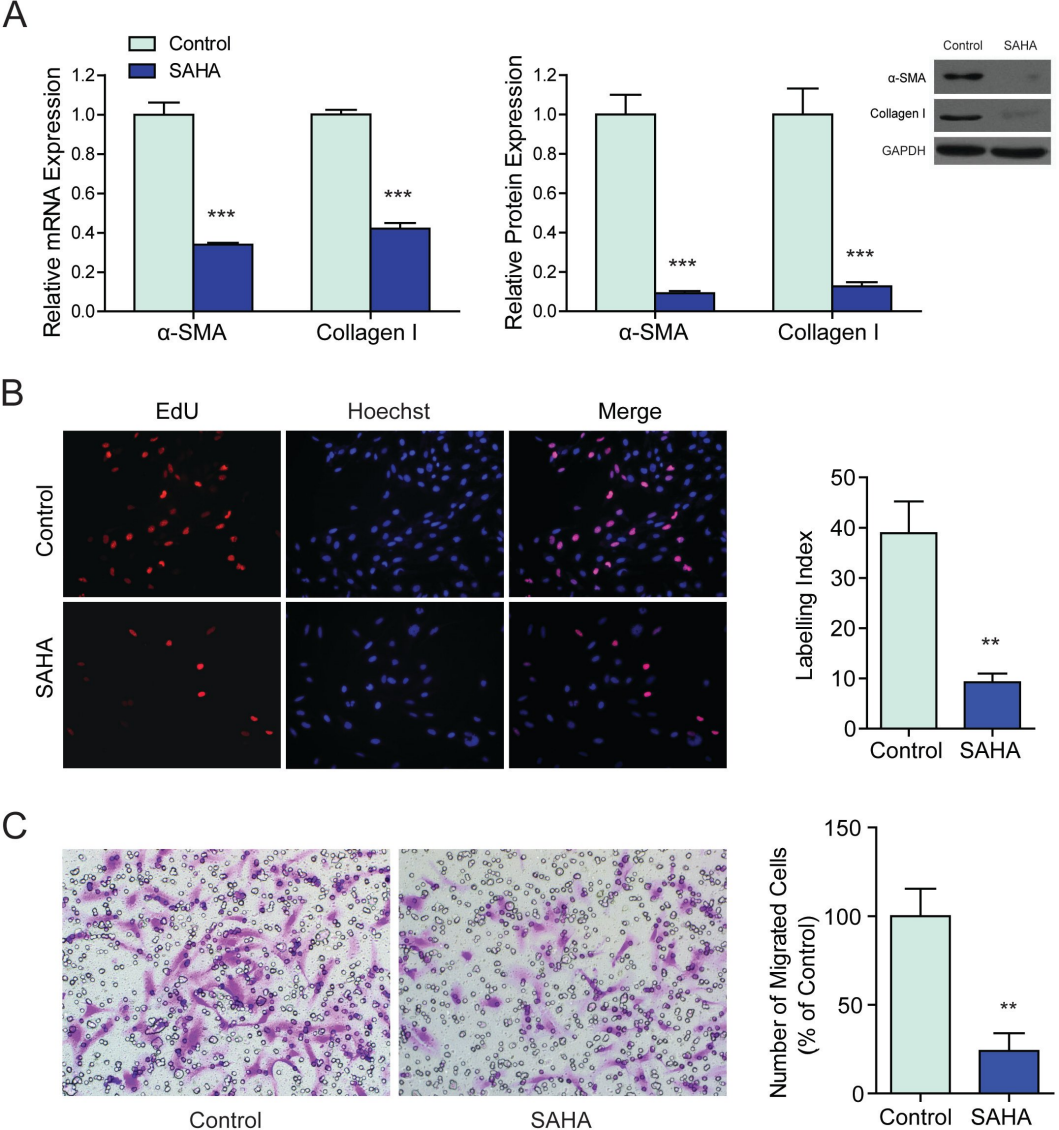
Suberoylanilide hydroxamic acid (SAHA) reversed the activation phenotype of a human hepatic stellate cell (HSC) line—LX2. (A) LX2 cells were exposed to 2.5 µM SAHA, the mRNA and protein expression of HSCs activation biomarkers (*α*-SMA and collagen I) were determined by real-time quantitative polymerase chain reaction and western blot, GAPDH was used as a loading control. (B) SAHA inhibited the proliferation of LX2 cells. EdU positive cells were detected by Alexa Fluor 594 azide (red); nuclei were counter stained with Hoechst 33258 (blue). Original magnification ×200. The EdU incorporation rate was expressed as Labeling Index according to the following formula: number of EdU positive nuclei × 100/number of total nuclei. (C) SAHA inhibited the migration of LX2 cells. Cells migrated through the transwell membrane were stained with crystal violet. Original magnification ×200. ^∗∗^*P* < 0.01, ^∗∗∗^*P* < 0.001, compared to the control.

Expression of *α*-SMA and collagen I, two widely used markers for HSCs activation, was examined in SAHA treated LX2 cells. By real-time RT-PCR, we found that LX2 cells cultured in the medium containing 2.5 µM SAHA for 24 h showed a decreased mRNA transcription of *α*-SMA and collagen I (Fig. 1A). The decreased expressions of *α*-SMA and collagen I at protein level were verified by western blots with LX2 cells treated by SAHA for 48 h (Fig. 1A). To investigate the effects of SAHA on HSCs activation, cell proliferation and migration were examined by EdU incorporation assay and trans-well assay respectively. The proliferation (Fig. 1B) and migration (Fig. 1C) were significantly inhibited when LX2 cells were treated with 2.5 µM SAHA for 48 h. These findings suggested that SAHA was able to inhibit HSCs activation efficiently.

### HMGB1 pathway was the one of the most important pathways that affected by SAHA in LX2 cells

The mechanisms underlying SAHA induced suppression of HSCs were explored by cDNA array analyses. cDNAs prepared from SAHA treated or untreated LX2 cells were hybridized to Affymetrix GeneChip Human Gene 1.0 ST Arrays, each with biological replica. The gene expression data have been deposited in a publicly accessible database (ArrayExpress, http://www.ebi.ac.uk/arrayexpress/, accession number E-MTAB-3764). The BRB-Array Tools were used for array data analysis and class comparison. Among the 24,557 genes detected, 504 genes were amplified with a differential expression ratio of 2.0 between SAHA treated and untreated LX2 cells. Out of the 504 genes, the expressions of 331 genes were up-regulated and 173 were down-regulated (Table S2).

In order to explore the biological significance of these differentially expressed genes, canonical pathway analyses were performed by IPA based on curated Ingenuity Pathways Knowledge Base. The top 10 pathways of up-regulated genes include: Remodeling of Epithelial Adherent Junctions, VDR/RXR (vitamin D receptor/retinoid X receptor) and PPAR (peroxisome proliferator-activated receptor) Signaling (Table S3). It has been evidenced that VDR/RXR signaling pathways (Li et al., 2002; Chen et al., 2004) and PPAR signaling pathways (Miyahara et al., 2000) were significantly suppressed during HSC activation, and both pathways were closely related to the proliferative phenotype of activated HSCs. The increased expression of genes involved in VDR/RXR signaling pathways and PPAR signaling pathways in SAHA treated LX2 cells suggested that SAHA might reverse the activation phenotype of HSCs through reactivation of VDR/RXR and PPAR signaling pathways.

**Figure 2:**
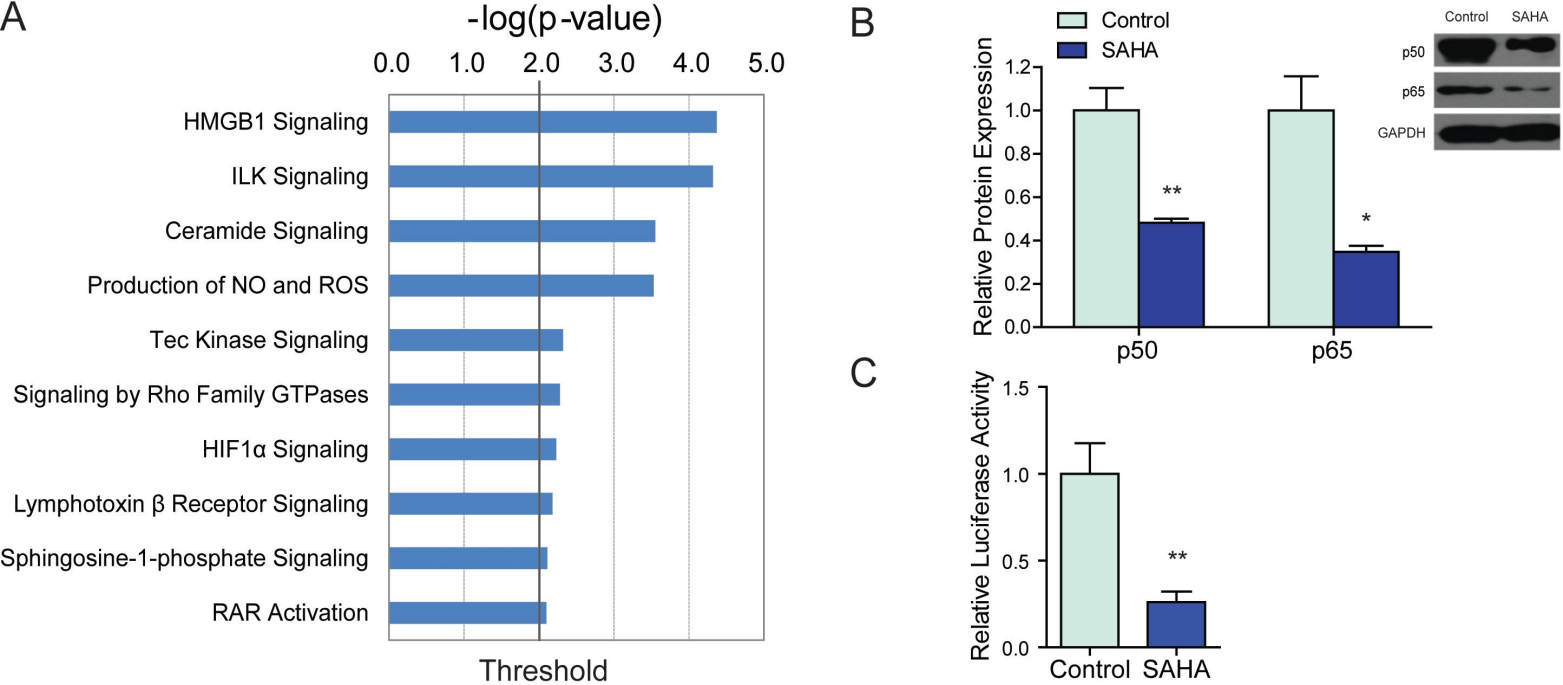
Canonical pathway analysis and further validation. (A) The top 10 canonical pathways of genes down-regulated by 2.5 µM SAHA in LX2 cells as determined by Ingenuity Pathway Analysis. The *x* axis showed the negative log of *p* value. (B) The protein expressions of p50 and p65 in 2.5 µM SAHA treated LX2 cells were determined by western blot. GAPDH was used as a loading control. (C) The activity of NF-*κ*B in SAHA treated LX2 cells were detected by a dual luciferase reporter system. NF-*κ*B driven firefly luciferase activity was assayed by a dual luciferase reporter system, renilla luciferase activity served as internal control, the results were expressed as relative luciferase activity. ^∗^*P* < 0.05, ^∗∗^*P* < 0.01, compared to the control.

The top 10 pathways of down-regulated genes were mostly related to inflammation, and some of them have been proved to promote HSCs activation or organ fibrosis, including: HMGB1 (high mobility group box 1 protein, HMGB1) Signaling (Li, Gao & Li, 2014; Wang et al., 2013), ILK (integrin—linked kinase, ILK) (Zhang et al., 2006) Signaling and Ceramide Signaling (Teichgraber et al., 2008) (Table S4 and Fig. 2A). HMGB1 pathway was the top pathway of down-regulated genes that contributed to the suppressive effects of SAHA on HSCs activation. Decreased genes in HMGB1 pathway include: NF-*κ*B1 (nuclear factor of kappa B1, 2.681-fold), RHOD (ras homolog family member D, 2.062-fold), RHOJ (ras homolog family member J, 4.348-fold), PIK3CG (phosphatidylinositol-4,5-bisphosphate 3-kinase, catalytic subunit gamma, 2.013-fold), KAT6B (K(lysine) acetyltransferase 6B, 2.174-fold) and TNFRSF11B (tumor necrosis factor receptor superfamily, member 11b, 3.047-fold). Although it has been evidenced that HMGB1 activated HSCs and exhibitd pro-fibrogenic effects, inhibition of HMGB1 expression by siRNA inhibited the synthesis of *α*-SMA and collagen in transfected HSCs (Ge et al., 2011) (see also Fig. S2), the expression of HMGB1 was not affected by SAHA. The inhibitory effects of SAHA on HSCs activation were not dependent on the direct inhibition of HMGB1 expression.

### SAHA induced down-regulation of NF-*κ*B1 is HMGB1 dependent

NF-*κ*B1 plays a key role in HSCs activation and liver fibrosis (Wang et al., 2014). It has been reported that HDAC inhibitors (such as TSA and valproic acid) can inhibit the activation of NF-*κ*B in malignant myeloblasts, although the exact mechanisms remain elusive (Fabre et al., 2008). We proposed that SAHA might attenuate HSCs activation through suppression of NF-*κ*B1. The protein expression and transcriptional activity of NF-*κ*B1 were examined in SAHA treated LX2 cells. By western blot, we confirmed that the expression of both p50, the mature subunit of NF-*κ*B1, and p65 which forms the most abundant heterodimeric p65-p50 complex were down-regulated by SAHA (Fig. 2B). By luciferase reporter assay, we found that the transcriptional activity of NF-*κ*B1 was also reduced by SAHA (Fig. 2C). Accordantly, in the study by Park et al. (2014), SAHA treatment reduced the phosphorylation of I*κ*B-*α* and NF-*κ*B p65, indicating suppressed NF-*κ*B activation by SAHA in HSCs.

**Figure 3:**
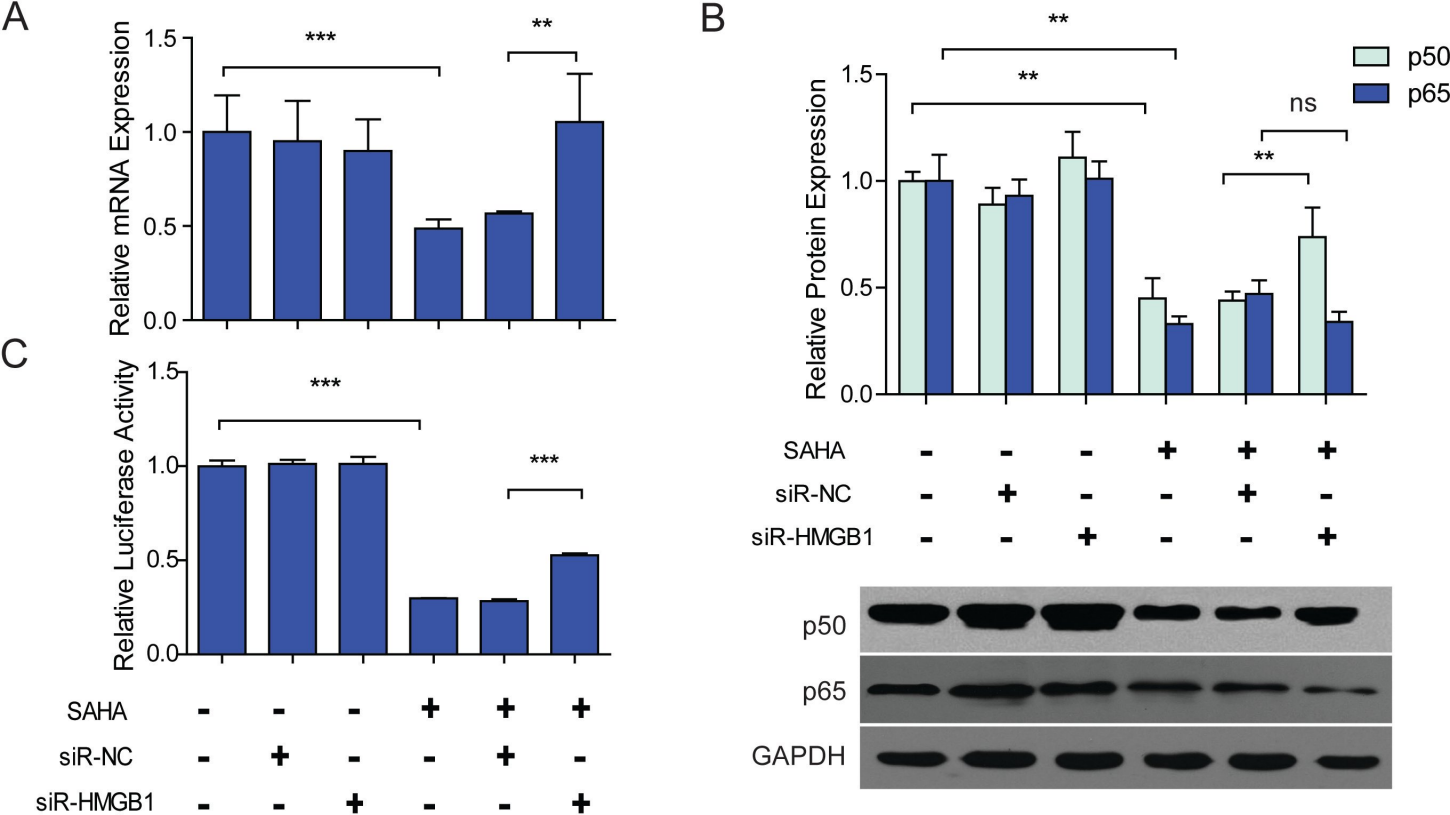
SAHA induced down-regulation of NF-*κ*B1 was HMGB1 dependent. LX2 cells were transfected by siR-NC or siR-HMGB1 48 h before 2.5 µM SAHA treatment. (A) The mRNA expression of NF-*κ*B1 (p50) in each group was detected by RT-PCR. (B) Protein expressions of p50 and p65 in each group were determined by western blot assay. (C) Dual luciferase reporter assay. The activity of NF-*κ*B in each group was detected by a dual luciferase reporter system. NF-*κ*B driven firefly luciferase activity was assayed by a dual luciferase reporter system, renilla luciferase activity served as internal control, the results were expressed as relative luciferase activity. The mRNA or protein expressions were normalized to GAPDH. ^∗∗^*P* < 0.01, ^∗∗∗^*P* < 0.001, ns, not significant, *P* > 0.05, compared to untreated control cells or cells transfected by siR-NC, respectively.

Although the expression of HMGB1 is not affected by SAHA, we found that the suppressive effects of SAHA on NF-*κ*B1 expression and activity were dependent on HMGB1. Silencing of HMGB1 (Fig. S2) did not affect the mRNA transcription of NF-*κ*B1, but when HMGB1 was silenced, the treatment of SAHA failed to induce the reduction of NF-*κ*B1 mRNA in LX2 cells (Fig. 3A). We further evidenced that knocking down of HMGB1 partially rescued the down-regulation of p50 protein by SAHA. After knocking down of HMGB1, the expression of p50 protein increased (1.63-fold) in SAHA treated LX2 cells , while the expression of p65 was not affected (Fig. 3B). Dual luciferase reporter assay showed similar results. After knocking down of HMGB1, the activity of NF-*κ*B increased significantly (1.76-fold) in SAHA treated LX2 cells (Fig. 3C). These data suggested that SAHA induced suppression of NF-*κ*B1 expression and activity were HMGB1 dependent.

HMGB1 can increase the binding affinity of many sequence-specific transcription factors to their cognate DNA, such as p53, p73, the retinoblastoma protein (Rb) and the estrogen receptor (Fiuza et al., 2003; Travers, 2003). HMGB1 is also important for the transcriptional activity of NF-*κ*B. It can enhance the binding of NF-*κ*B to its target sequences and is necessary for NF-*κ*B dependent target mRNA expression (Luan et al., 2010). Moreover, in TNF-alpha-stimulated fibroblasts, the predominant form of NF-*κ*B is the p65/p50 heterodimer (Luan et al., 2010), whose DNA binding affinity is indeed enhanced by HMGB1. However, it is the first time we reported here that the mRNA transcription of NF-*κ*B itself is also dependent on the presence of HMGB1.

### SAHA treatment changed the acetylation of intracellular HMGB1

**Figure 4:**
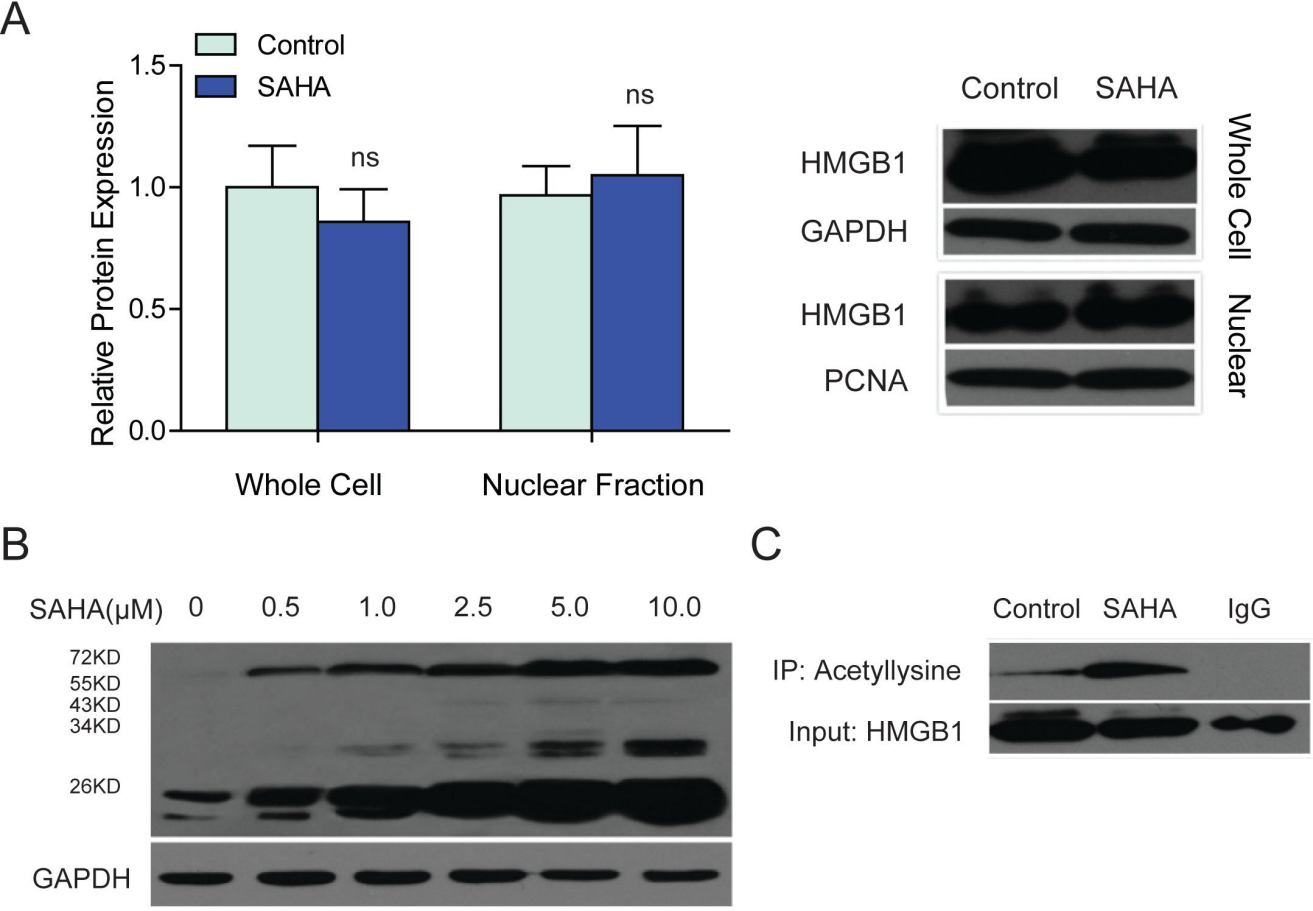
SAHA treatment improved the lysine acetylation of intracellular HMGB1. (A) The protein level of HMGB1 in the whole cell or nuclear lysates of SAHA treated LX2 cells was detected by western blot, ns, not significant, *P* > 0.05. GAPDH was used as a loading control for the whole cell lysates, and PCNA were used as a loading control for the nuclear fraction. (B) The lysine acetylation levels of total proteins from SAHA-treated LX2 cells were analyzed by western blot. The concentrations of SAHA were 0.5 µM, 1.0 µM, 2.5 µM, 5.0 µM and 10.0 µM as indicated. GAPDH was used as a loading control. (C) Lysine acetylation of HMGB1 was detected by immunoprecipitation. IgG was served as a negative control.

According to the cDNA array data, in SAHA treated LX2 cells, the abundance of HMGB1 mRNA was not changed. We further evidenced that the protein expression of HMGB1 was not affected either (Fig. 4A). We then asked how HMGB1 was involved in SAHA induced suppression of NF-*κ*B1.

As we know, HMGB1 is among the most important chromatin proteins. In the nucleus, HMGB1 interacts with nucleosomes, transcription factors, and histones; it organizes the DNA and regulates transcription (Park et al., 2003; Yang et al., 2013). HMGB1 can be modified by acetylation, its function also depend on the number of acetylated lysine residues (Ugrinova, Pashev & Pasheva, 2009). We wondered that if SAHA made any posttranslational modification and altered the biofunction of HMGB1?

The acetylation of HMGB1 was examined by immunoprecipitation. The treatment of SAHA enhanced lysine acetylation of both histone and nonhistone proteins in a dose-dependent manner (Fig. 4B). The acetylation of HMGB1 was at a very low level in untreated LX2 cells. After 48 h treatment with SAHA, the acetylation of HMGB1 increased dramatically (Fig. 4C).

It has been reported that acetylation prevents HMGB1 from interacting with the nuclear-importer protein complex, so re-entry to the nucleus is blocked. Acetylated HMGB1 tends to translocate into cytoplasm and subsequently migrates to cytoplasmic secretory vesicles for release into the extracellular space (Lu et al., 2014; Yang et al., 2013).

To study the possible impact of SAHA treatment on the distribution of intracellular HMGB1 protein, western blot of HMGB1 with whole cells or nuclear lysates of LX2 cells and immunofluorescent staining of HMGB1 in SAHA treated LX2 cells were performed. By western blot, there was no detectable change of the HMGB1 in the nuclei of SAHA treated LX2 cells (Fig. 4A). Consistently, we did not find obvious nucleus-cytoplasm translocation of HMGB1 in SAHA treated LX2 cells by immunofluorecence. Actually, HMGB1 protein was mainly distributed in the cytoplasm of SAHA treated LX2 cells as well as untreated LX2 cells (Fig. S3).

Knocking down of HMGB1 did not affect the expression and activity of NF-*κ*B1. But HMGB1 was required for SAHA induced suppression of NF-*κ*B1. The only detectable change of HMGB1 in SAHA treated LX2 cells was the increased acetylation of HMGB1. So we proposed that increased acetylation of intracellular HMGB1 was most likely to be the underlying mechanism of SAHA induced down-regulation of NF-*κ*B1. However, only HMGB1 knockdown experiments were performed in the present study, further studies are needed to test the activity of NF-*κ*B1 and HSCs activation after HMGB1 overexpression, and to clarify this very interesting and complicated epigenetic mechanisms involved in the anti-fibrotic effects of HDACi.

## Conclusions

In the present study, we found that SAHA, a clinically applicable class I and II HDAC inhibitor, was able to reverse HSCs activation. The underlying mechanisms were explored by transcriptomic profiling and subsequent bioinformatics analyses. HMGB1 pathway turned out to be one of the most important pathways that contributed to the inhibitory effects of SAHA on HSCs activation. We verified that HMGB1 played a decisive role in SAHA induced inhibition of p50 expression and NF-*κ*B’s activity. Acetylation of HMGB1 by SAHA might be responsible for suppressed expression and function of NF-*κ*B1. Because no obvious nuclear to cytosol translocation of HMGB1 has been observed, how SAHA induced acetylation of cytoplasmic HMGB1 is related to its biofunction and causes suppression of NF-*κ*B1 deserves further investigation. We should also keep in mind that the findings reported here were based on a human HSC cell line, it is important to confirm the present findings in primary HSCs or *in vivo* model (CCl4 or TAA-induced liver fibrosis) in future studies.

## Supplemental Information

10.7717/peerj.1362/supp-1 Table S1 siRNA sequences and primers for RT-PCR

10.7717/peerj.1362/supp-2 Table S2 Differentially expressed genes in SAHA treated LX2 cells

10.7717/peerj.1362/supp-3 Table S3 Top 10 Canonical Pathways of Up-regulated Genes in SAHA treated LX2 cells

10.7717/peerj.1362/supp-4 Table S4 Top 10 Canonical Pathways of Down-regulated Genes in SAHA treated LX2 cells

10.7717/peerj.1362/supp-5 Figure S1 The IC50 of SAHA for LX2 cells (A) LX2 cells were exposed to different concentrations of SAHA ranging from 0 to 10 µM for 0 h, 24 h, 36 h 48 h or 72 h. Cell proliferation was determined by Cell Counting Kit-8. The optical density readings at 450 nm were determined by a microplate reader. The inhibition rate of SAHA was determined by comparison with the vehicle control. (B) The inhibitory rate of SAHA to the growth of LX2 cells at 48 h.

10.7717/peerj.1362/supp-6 Figure S2 Inhibition of HMGB1 expression by siRNA inhibits the synthesis of *α*-SMA and collagen I in transfected HSCs LX2 cells were transfected with HMGB1 specific siRNA (siR-HMGB1) or with negative control siRNA (siR-NC), their mRNA (A) and protein (B) expression levels were determined by real-time quantitative polymerase chain reaction or western blot after 24 h or 48 h, respectively. (C) The mRNA expression of *α*-SMA and collagen I in LX2 cells transfected with siR-HMGB1 or with siR-NC were determined by real-time quantitative polymerase chain reaction after 24 h, GAPDH was used as housekeeping gene. ^∗∗^*P* < 0.01, ^∗∗∗^*P* < 0.001, compared with siR-NC.

10.7717/peerj.1362/supp-7 Figure S3 The expression and distribution of HMGB1 in SAHA treated LX2 cells were deteced by immunofluorecence Original magnification × 400.

## Abbreviations

CCK8: Cell Counting Kit-8
ECM: Extracellular matrix
EdU: 5-ethynyl-20-deoxyuridine
HDACi: Histone deacetylases inhibitor
HMGB1: High mobility group box 1 protein
HSCs: Hepatic stellate cells
IC50: 50% inhibitory concentration
IPA: Ingenuity Pathways Analysis;
NF-*κ*B1: Nuclear factor kappa B1
PVDF: Polyvinyldifluoride
RT-PCR: Reverse transcription-PCR
SAHA: Suberoylanilide hydroxamic acid
siRNA: Small interfering RNA
siR-NC: Non-targeting control siRNA
TSA: Trichostatin A
*α*-SMA: *α*-smooth muscle actin

## Additional Information and Declarations

### Microarray Data Deposition

The following information was supplied regarding the deposition of microarray data: ArrayExpress, accession number E-MTAB-3764: http://www.ebi.ac.uk/arrayexpress/.
