# Supplementary figures and images for: Suberoylanilide hydroxamic acid suppresses hepatic stellate cells activation by HMGB1 dependent reduction of NF-κB1
Source: PeerJ. 2015 Nov 3;3:e1362. doi: 10.7717/peerj.1362 (PMC4636417; doi:10.7717/peerj.1362)

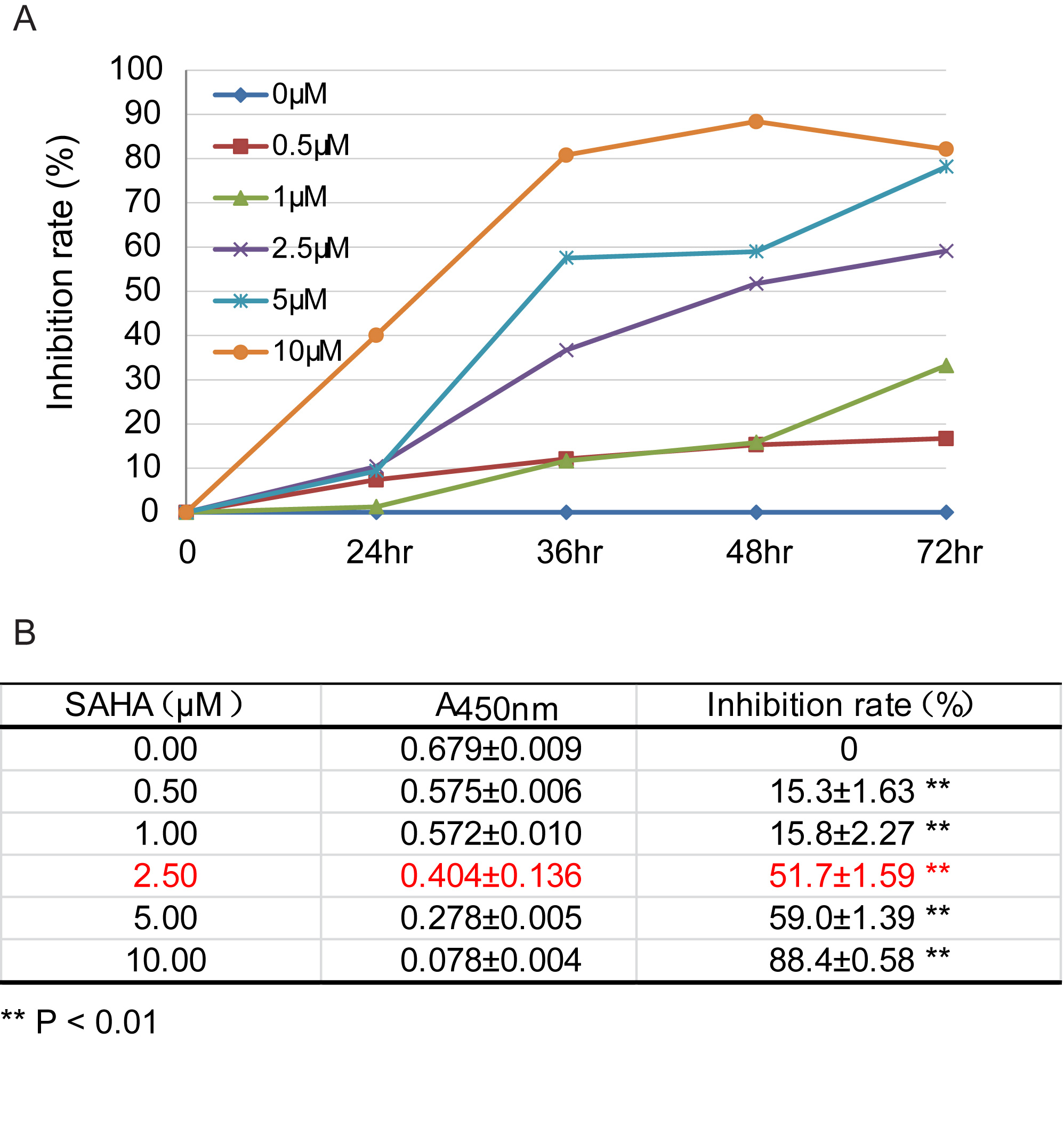

Supplement: Figure S1 — (A) LX2 cells were exposed to different concentrations of SAHA ranging from 0 to 10 µM for 0 h, 24 h, 36 h 48 h or 72 h. Cell proliferation was determined by Cell Counting Kit-8. The optical density readings at 450 nm were determined by a microplate reader. The inhibition rate of SAHA was determined by comparison with the vehicle control. (B) The inhibitory rate of SAHA to the growth of LX2 cells at 48 h. [file peerj-03-1362-s005.jpg]

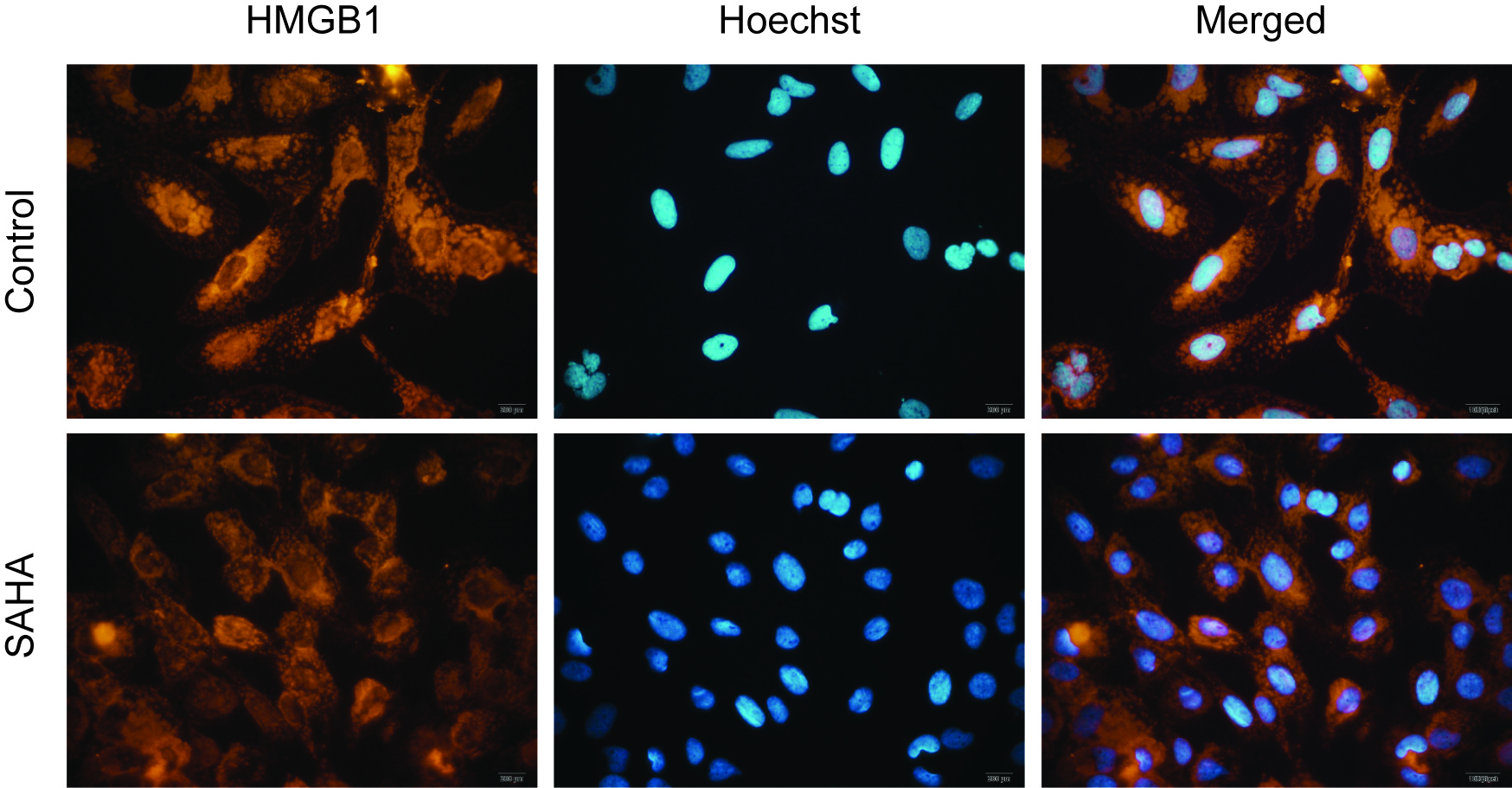

Supplement: Figure S2 — LX2 cells were transfected with HMGB1 specific siRNA (siR-HMGB1) or with negative control siRNA (siR-NC), their mRNA (A) and protein (B) expression levels were determined by real-time quantitative polymerase chain reaction or western blot after 24 h or 48 h, respectively. (C) The mRNA expression of α-SMA and collagen I in LX2 cells transfected with siR-HMGB1 or with siR-NC were determined by real-time quantitative polymerase chain reaction after 24 h, GAPDH was used as housekeeping gene. ∗∗P < 0.01, ∗∗∗P < 0.001, compared with siR-NC. [file peerj-03-1362-s006.jpg]

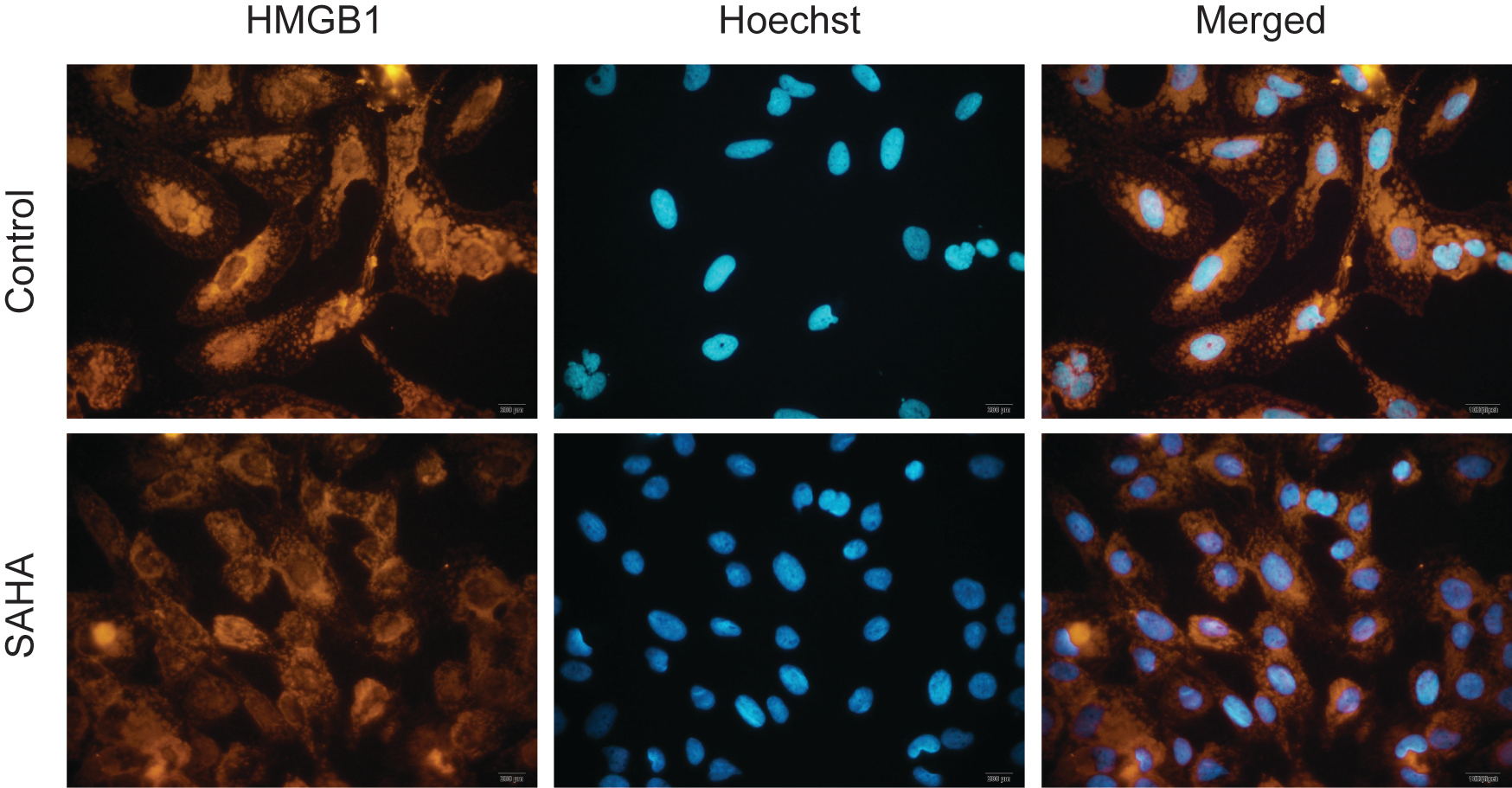

Supplement: Figure S3 — Original magnification × 400. [file peerj-03-1362-s007.jpg]
